# 2488 Parental perspectives on factors affecting participation in family centered rounds: Impact of technology use in trainee presentations

**DOI:** 10.1017/cts.2018.258

**Published:** 2018-11-21

**Authors:** Alexander Glick, Gabrielle Gold-von Simson, Michael Goonan, Diana Sandmeyer

**Affiliations:** 1 H+H Clinical and Translational Science Institute, NYU; 2 NYU Langone Health; 3 NYU School of Medicine

## Abstract

OBJECTIVES/SPECIFIC AIMS: Family centered rounding on pediatric inpatient units improves communication and family satisfaction. While the use of electronic medical record based devices and resources as part of the rounding process allows for immediate access of information and real time order management, there is limited data concerning parental perspectives’ about technology use on rounds, and about factors affecting participation during family centered rounds more generally. Our objectives were to examine parental (1) perspectives on factors that affect their participation during family centered rounds and (2) resource preference (tablet, computer on wheels, paper notes) used by trainees and the reasons for said preference. METHODS/STUDY POPULATION: We performed a cross-sectional study with English-speaking parents who were present for multidisciplinary family centered rounds and whose children were admitted to the inpatient pediatric unit at a tertiary care academic medical center. Parents were surveyed after rounds to ascertain their opinions on factors affecting their participation in rounds, preferences in respect to the resource used by trainee, and whether they believed the resource used on rounds that day affected their understanding or participation in rounds. Parents were also asked to articulate the reasons behind their preferred resource. Responses were analyzed using descriptive statistics, and qualitative responses were analyzed for themes. RESULTS/ANTICIPATED RESULTS: In total, 40 parents enrolled. Common responses regarding factors affecting parental participation included: information was explained in way that was easy to understand (90%), parents’ understanding of the medical information (85%), eye contact with the medical team (78%), if the medical team asks for parent input (75%), and the health of the child (70%). Fewer parents (23%) believed that the type of resource used affected their participation. Tablets were the preferred technology resource (33%) due to their portability, ease of accessing information, and that they encouraged interaction with the patient. Fewer preferred computers on wheels (27%) and paper notes (5%). In total, 35% of parents reported no preferred resource. No parents said that tablets were their least preferred resource. Reasons computers on wheels were least preferred (13% of parents) included their large size and that they limited eye contact, whereas, parents stated that paper notes were least preferred (13% of parents) because they were old-fashioned, easy to lose, and not accurate; 68% of parents stated the resource used did not affect their understanding on rounds that day, and 83% asserted the resource had no effect on participation. DISCUSSION/SIGNIFICANCE OF IMPACT: Clear and engaging communication during family centered rounds is most important to parents’ participation. The type of technology resource used is less relevant, but parents favor the use of tablets when they report a preference. Given the convenience for providers, tablet utilization as part of a family centered, trainee based rounding process has potential benefit.

